# Estrogen receptor alpha (ERα/ESR1) mediates the p53-independent overexpression of MDM4/MDMX and MDM2 in human breast cancer

**DOI:** 10.18632/oncotarget.7533

**Published:** 2016-02-20

**Authors:** Wendy M. Swetzig, Jianmin Wang, Gokul M. Das

**Affiliations:** ^1^ Department of Pharmacology and Therapeutics, Roswell Park Cancer Institute, Buffalo, NY, USA; ^2^ Department of Molecular Pharmacology and Cancer Therapeutics, The University at Buffalo, State University of New York, Buffalo, NY, USA; ^3^ Department of Bioinformatics and Biostatistics, Roswell Park Cancer Institute, Buffalo, NY, USA

**Keywords:** estrogen receptor alpha (ERα/ESR1), MDM2/HDM2, MDM4/MDMX/HDMX/HDM4, p53, breast cancer

## Abstract

MDM2 and MDM4 are heterodimeric, non-redundant oncoproteins that potently inhibit the p53 tumor suppressor protein. MDM2 and MDM4 also enhance the tumorigenicity of breast cancer cells in *in vitro* and *in vivo* models and are overexpressed in primary human breast cancers. Prior studies have characterized Estrogen Receptor Alpha (ERα/ESR1) as a regulator of MDM2 expression and an MDM2- and p53-interacting protein. However, similar crosstalk between ERα and MDM4 has not been investigated. Moreover, signaling pathways that mediate the overexpression of MDM4 in human breast cancer remain to be elucidated. Using the Cancer Genome Atlas (TCGA) breast invasive carcinoma patient cohort, we have analyzed correlations between ERα status and MDM4 and MDM2 expression in primary, treatment-naïve, invasive breast carcinoma samples. We report that the expression of MDM4 and MDM2 is elevated in primary human breast cancers of luminal A/B subtypes and associates with ERα-positive disease, independently of p53 mutation status. Furthermore, in cell culture models, ERα positively regulates MDM4 and MDM2 expression via p53-independent mechanisms, and these effects can be blocked by the clinically-relevant endocrine therapies fulvestrant and tamoxifen. Additionally, ERα also positively regulates p53 expression. Lastly, we report that endogenous MDM4 negatively regulates ERα expression and forms a protein complex with ERα in breast cancer cell lines and primary human breast tumor tissue. This suggests direct signaling crosstalk and negative feedback loops between ERα and MDM4 expression in breast cancer cells. Collectively, these novel findings implicate ERα as a central component of the p53-MDM2-MDM4 signaling axis in human breast cancer.

## INTRODUCTION

MDM2/HDM2 and MDM4/MDMX/HDMX are homologous, heterodimeric, E3-ubiquitin protein ligases that are classically known for their non-redundant abilities to inhibit the p53 tumor suppressor protein (reviewed in [1–4]) p53 regulates a variety of cellular processes, including DNA repair, apoptosis, cell cycle arrest, senescence, autophagy and metabolism, amongst others (reviewed in [5–9]). MDM2 and MDM4 constrain p53 function by binding to and inhibiting the transactivation domain of p53, by cooperating to poly-ubiquitinate p53, and by facilitating the translocation of p53 to the cytosol [10–30]. p53 is also known for its ability to upregulate *MDM2* and *MDM4* gene expression through an auto-inhibitory negative feedback loop [31–37]. However, p53-independent mechanisms by which *MDM2* and *MDM4* gene expression are regulated remain poorly understood.

*MDM2* is an established oncogene in breast cancer. *In vitro*, MDM2 enhances the proliferation of human breast cancer cells and antagonizes apoptosis [38–43]; *in vivo, Mdm2* transgene expression initiates mammary gland tumorigenesis in murine models [44]; and in breast cancer patients, MDM2 protein overexpression and *MDM2* gene amplification are associated with decreased overall and/or disease-free survival [45–48]. Consistent with its oncogenic role, the *MDM2* gene is overexpressed at the mRNA and protein levels in 26-73% of primary human breast cancers [47, 49–54]. Since *MDM2* gene amplifications are relatively infrequent [45, 52, 53, 55–58], the overexpression of MDM2 in breast cancer is likely mediated by aberrant gene regulation.

Estrogen receptor alpha/estrogen receptor 1 (ERα/ESR1) is a nuclear hormone receptor and oncoprotein that is expressed in approximately 70% of breast cancers [59, 60]. Interestingly, MDM2 expression positively correlates with ERα expression in primary human breast tumors and human breast cancer cell lines, and ERα has been proposed to upregulate MDM2 expression [38, 50, 51, 56, 61–68]. In addition, MDM2 also forms a protein complex with ERα and facilitates the ubiquitination and degradation of ERα [41, 43, 66, 69]. This establishes a negative feedback loop between MDM2 and ERα. However, the ability of ERα and MDM4 to similarly interact with one another and to regulate one another's expression remains to be elucidated.

Like MDM2, MDM4 also plays a protumorigenic role in human breast cancer cells that are cultured *in vitro* or *in vivo* as murine xenografts [39, 40, 55, 70–72]. Knockdown of MDM4 inhibits the proliferation of breast cancer cells, induces the expression of the cyclin dependent kinase inhibitor CDKN1A/p21^waf1/cip1^, and causes G_1_-phase cell cycle arrest and senescence [39, 40, 55, 70]. Additionally, loss of MDM4 reduces cell viability, sensitizes cells to agent-induced apoptosis and upregulates p53 in breast cancer cell culture models [30, 39, 40, 71]. MDM4 also cooperates with MDM2 to facilitate the ubiquitination of p53 in breast cancer cells [30]. In the clinic, these protumorigenic functions of MDM4 are likely facilitated by the overexpression of the *MDM4* gene, which occurs in approximately 20-55% of primary human breast tumors [49, 53, 55, 70]. However, mechanisms that mediate the overexpression of MDM4 in breast cancer have not been identified, and factors capable of regulating *MDM4* gene expression in human cells remain largely unknown, with only two main pathways having been identified to date: p53 and mitogen-activated protein kinase (MAPK) [35, 73]. Since MDM4 expression is frequently elevated in luminal breast cancers [55], and the majority of luminal tumors are ERα-positive [74], we propose that ERα and MDM4 may be coexpressed with one another in human breast cancer and may regulate each other's expression.

The objective of the present study is to examine signaling crosstalk between ERα, MDM4, MDM2 and p53 in human breast cancer at the levels of gene expression and protein-protein interactions. We have used treatment-naive primary human breast carcinomas with corresponding gene expression data from the Cancer Genome Atlas invasive breast carcinoma (TCGA BRCA) patient cohort, as well as complementary cell culture models, to demonstrate that ERα mediates the overexpression of *MDM4* and *MDM2* genes in human breast cancer. We also provide evidence that, like MDM2, MDM4 forms a protein complex with ERα and negatively regulates ERα expression, thereby establishing a negative feedback loop between ERα and MDM4/MDM2 at the expression level.

## RESULTS

### *MDM4* and *MDM2* mRNA expression is elevated in ERα-positive primary breast invasive carcinoma samples

**Figure 1:**
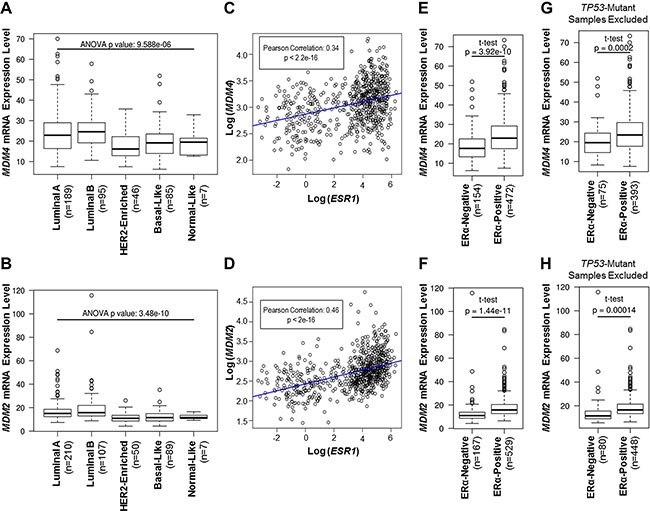
*MDM4* and *MDM2* mRNA expression is elevated in ERα-positive primary breast invasive carcinoma samples (**A**–**H**) Analyses of *MDM4* or *MDM2* mRNA expression in TCGA breast invasive carcinoma cohort. (**A, B**) Tumor types are classified according to the PAM50 model of intrinsic subtype classification. (**C, D**) Pearson product-moment correlation coefficient test analysis of correlations between *ESR1/ERα* mRNA and either *MDM4* mRNA (panel C) or *MDM2* mRNA (panel D). (**E, F**) Tumor types are classified according to ERα status. (**G, H**) As in panels E and F, but *TP53*-mutant tumors were excluded from the analysis to study gene expression patterns that were independent of *TP53* mutations. Note that for panels A, C, E and G, samples with *MDM4* gene amplifications were excluded from the analysis to study patterns in *MDM4* mRNA expression that were independent of *MDM4* gene amplification. For panels B, D, F and H, samples with *MDM2* gene amplifications were excluded from the analysis to study patterns in *MDM2* mRNA expression that were independent of *MDM2* gene amplification.

It is well-established that MDM2 protein is coexpressed with ERα in human breast cancer cell lines and primary human breast tumors [50, 51, 56, 62, 63, 68]. However, it is not known if *MDM2* mRNA expression also correlates with ERα expression in primary breast tumors. Moreover, analyses of correlations between the MDM2 homolog, MDM4, and ERα expression have not been performed. In the present study, we utilized The Cancer Genome Atlas invasive breast carcinoma (TCGA BRCA) RNA-Seq gene expression dataset to analyze the expression of *MDM4* and *MDM2* mRNA in treatment-naive primary human breast invasive carcinoma samples. The samples had been previously categorized according to ERα status and intrinsic molecular subtype using the PAM50 50-gene subtype predictor model [75, 76]. To study *MDM4* and *MDM2* mRNA expression patterns that were independent of *MDM4* and *MDM2* gene amplification, samples that were known to have *MDM4* or *MDM2* gene amplifications (as described in [75]) were excluded from our *MDM4* and *MDM2* gene expression analyses, respectively. We observed that *MDM4* and *MDM2* mRNA expression was elevated in primary breast invasive carcinomas of luminal A and luminal B molecular subtypes, as compared to HER2-enriched, basal, and normal-like subtypes (Figure 1, panels A and B). Notably, luminal A and luminal B subtypes are enriched for ERα-positive tumors, as compared to HER2-enriched, basal, or normal-like subtypes [74], indicating that *MDM4* and *MDM2* gene expression may correlate with ERα expression. Indeed, Pearson product-moment correlation coefficient tests identified significant positive correlations between *ERα/ESR1* and *MDM4* mRNA expression (Figure 1C) and between *ERα/ESR1* and *MDM2* mRNA expression (Figure 1D). In addition, *MDM4* and *MDM2* mRNA expression was significantly higher in ERα-positive tumors, as compared to ERα-negative tumors (Figure 1, panels E and F). These findings establish a co-expression pattern for ERα and MDM4, as well as for ERα and MDM2, in primary human invasive breast carcinomas. Furthermore, this correlation is independent of *MDM4* and *MDM2* gene amplifications, suggesting that mechanisms other than gene amplification govern the upregulation of *MDM4* and *MDM2* mRNA in ERα-positive tumors.

Due to the well-characterized roles of MDM4 and MDM2 in the p53 pathway, we also measured *MDM4* and *MDM2* gene expression in terms of *TP53* mutation status. We observed that *MDM4* and *MDM2* mRNA expression was elevated in *TP53* wild type tumors, as compared to *TP53* mutant tumors (Supplementary Figure S1), consistent with wild type p53 being a positive transcriptional regulator of *MDM4* and *MDM2* genes [31–37]. It is known that the majority of *TP53*-mutant breast tumors are ERα-negative [77], and our analyses have revealed that ERα-negative tumors have reduced expression of *MDM4* and *MDM2* mRNA (Figure 1, panels E and F). Therefore, we tested if our observed positive correlations between ERα and either *MDM4* or *MDM2* gene expression were dependent upon *TP53* mutation status. To do this, we excluded samples with *TP53* mutations and analyzed *MDM4* and *MDM2* gene expression in the *TP53* wild type group. This analysis revealed that *MDM4* and *MDM2* mRNA expression was still higher in the ERα-positive tumors than in the ERα-negative tumors, even after exclusion of the *TP53* mutant tumors (Figure 1, panels G and H). This finding indicates that the associations between ERα and either *MDM4* or *MDM2* gene expression are independent of *TP53* mutations. As such, we postulated that ERα may regulate MDM4 and MDM2 expression via p53-independent mechanisms.

### Loss of ERα and/or inhibition of ERα downregulates MDM4 and MDM2 in breast cancer cells

**Figure 2:**
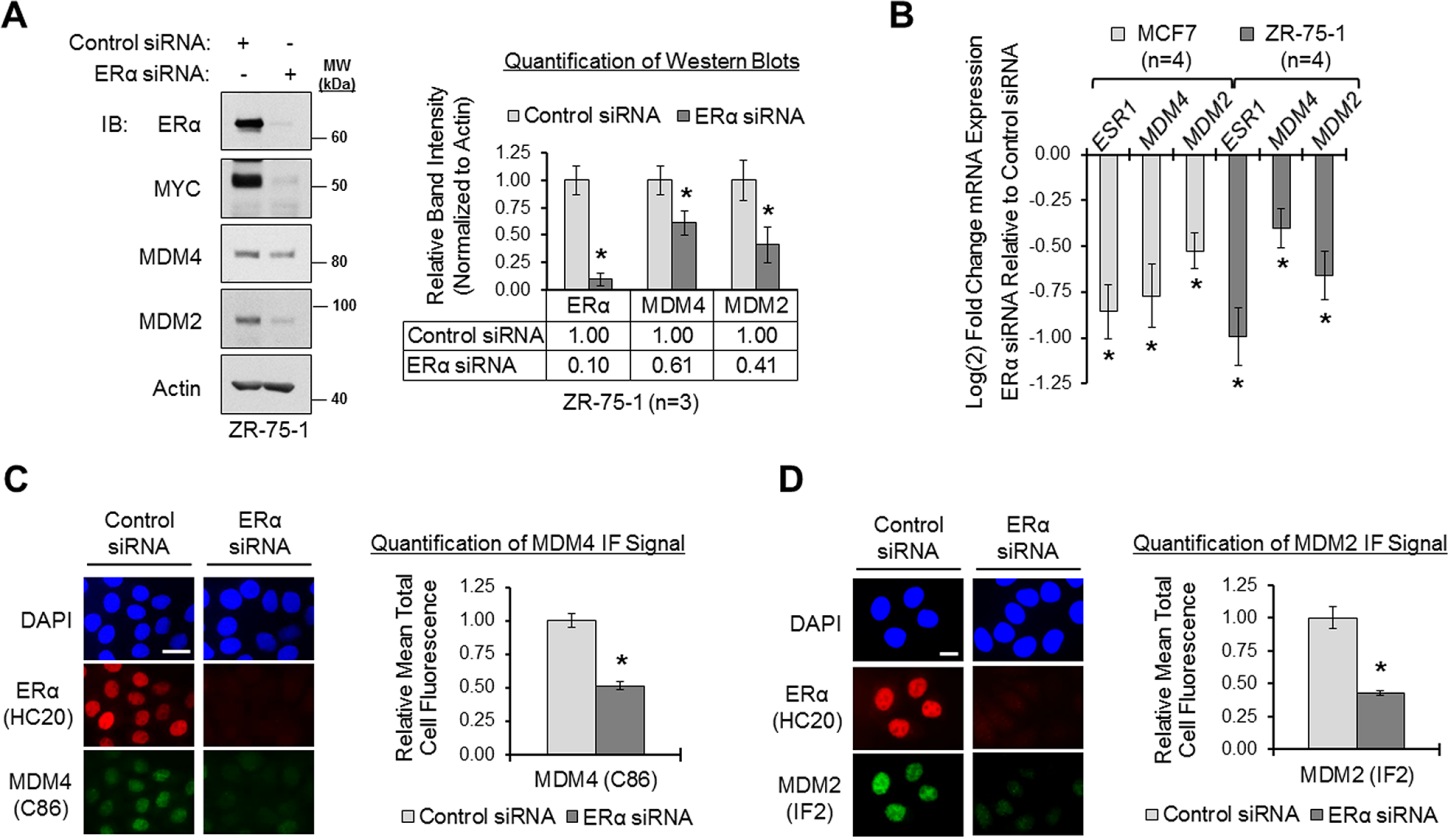
Loss of ERα downregulates MDM4 and MDM2 in breast cancer cells (**A**) Western blot analysis of ZR-75-1 cells transfected with control or ERα siRNA (sequence #1) for 22 h. Left panel: representative blot. Right panel: quantification of western blots. Error bars = SEM of biological replicates (*n* = 3). (**B**) qPCR analysis of MCF7 and ZR-75-1 cells transfected with control or ERα siRNA for 22 h. *ESR1/ERα*, *MDM4* and *MDM2* mRNA expression values were normalized to *ACTB*, and ERα siRNA-transfected samples were relatively compared to control samples according to the ΔΔ*C*_q_ method. Control samples are set at 0 on the y-axis. Fold change values are log_2_ transformed to ensure an accurate representation of values less than 1; therefore, a change of 1 unit on the log_2_ scale would be equivalent to a 2-fold change in expression on a linear scale. Error bars = SEM of biological replicates (*n* = 4). (**C**) Immunofluorescent (IF) microscopy of ERα and MDM4 in MCF7 cells that were transfected with control or ERα siRNA for 22 h. Nuclei were labeled with DAPI. Exposure times were equal for control and ERα siRNA-transfected cells. Left panel: representative image. Right panel: quantification of MDM4 IF signal. Error bars = SEM of total cell fluorescence (*n* = 15 cells from 3 independent coverslips). (**D**) IF microscopy of ERα and MDM2 in MCF7 cells that were transfected with control or ERα siRNA for 22 h. Nuclei were labeled with DAPI. Exposure times were equal for control and ERα siRNA-transfected cells. Left panel: representative image. Right panel: quantification of MDM2 IF signal. Error bars = SEM of total cell fluorescence (*n* = 15 cells from 3 independent coverslips). *p < 0.05, unpaired *t*-test. Microscopy scale bar = 20 μm. DAPI = 4›,6-diamidino-2-phenylindole. IB = immunoblot. kDa = kilodaltons. MW = molecular weight. SEM= standard error of the means.

Since ERα-positive primary invasive breast carcinomas have elevated expression of *MDM4* and *MDM2* genes, we hypothesized that ERα mediates the upregulation of MDM4 and MDM2 in human breast cancer. Previous reports have proposed that ERα modulates MDM2 expression [38, 50, 61, 62, 64–68]. However, it is not known if ERα also regulates MDM4 expression. We used the luminal human breast adenocarcinoma cell lines ZR-75-1 and MCF7 as *in vitro* model systems of ERα-positive breast cancer, and we investigated how loss of ERα expression affected *MDM4* gene expression at the protein and mRNA levels, as compared to its effects on *MDM2* gene expression. As shown in Figure 2, panel A, we used ERα siRNA to efficiently knock down ERα expression by approximately 90% in ZR-75-1 cells, which resulted in the decreased expression of MYC, a well-characterized ERα-target gene [78, 79] shown here as a positive control. Notably, when ERα was knocked down, we also observed significant reductions in MDM4 and MDM2 protein expression, by approximately 40% and 60%, respectively, demonstrating that ERα positively regulates the expression of both MDM4 and MDM2 (Figure 2A). Substantial decreases in MDM4 and MDM2 protein expression were also observed using a second ERα siRNA and a second cell line, MCF7, demonstrating the specificity of the effect (Supplementary Figure S2, panel A). qPCR analysis of *MDM4* and *MDM2* mRNA expression in MCF7 and ZR-75-1 cells that had been transfected with ERα siRNA or non-silencing control siRNA revealed that loss of ERα also resulted in a significant downregulation of *MDM4* and *MDM2* mRNA expression (Figure 2B). This finding is in agreement with our data from Figure 1, where we observed that ERα expression positively correlated with *MDM4* and *MDM2* mRNA expression in primary breast carcinomas. Therefore, we conclude that ERα is a positive regulator of not only *MDM2* gene expression, but also of *MDM4* gene expression.

Having demonstrated that ERα regulates *MDM4* and *MDM2* mRNA expression (Figure 2B), and additionally, that *MDM4* and *MDM2* mRNA expression is specifically elevated in ERα-positive primary human breast tumors (Figure 1), we postulated that ERα transcriptionally regulates *MDM4* and *MDM2* genes. We mined 8 different publicly-available ERα ChIP-Seq and ChIP-Chip datasets from the Nuclear Receptor Cistrome database [80] and the ENCODE Project database [81], where the ERα ChIP was performed in p53 wild type MCF7 human breast cancer cells or p53 mutant T-47D human breast cancer cells. We used Integrated Genomics Viewer (IGV) software to visualize the binding of ERα to the *MDM4* and *MDM2* genes in these ChIP datasets. Consensus estrogen receptor binding sites (ERBS) within the *MDM4* and *MDM2* genes were identified as regions that were bound by ERα in at least 2 or more of the 8 ChIP datasets. We observed 4 consensus ERBS within a downstream regulatory element of the *MDM4* gene (Supplementary Figure S3); likewise, we similarly observed 4 consensus ERBS within an upstream regulatory element of the *MDM2* gene (Supplementary Figure S4). To determine if these consensus ERBS were directly bound by ERα, we analyzed their DNA sequences for the presence of putative estrogen response element (ERE) sequences and/or ERE half sites using Dragon ERE Finder software [82]. We found that each of the consensus ERBS that we identified in both the *MDM4* and *MDM2* genes contained one or more ERE or half site (Supplementary Tables 1 and 2), indicating that ERα has the capability to directly bind to these regions.

Additionally, as further supportive evidence to demonstrate that ERα directly regulates *MDM4* and *MDM2* gene expression, we assessed the binding of the nuclear hormone receptor-associated pioneer transcription factor, forkhead box protein A1 (FOXA1). FOXA1 binding has previously been demonstrated to occur within close proximity to EREs, to predict the genomic locations of functional EREs, and to determine ERα-binding specificity [83-86]. We found that FOXA1 ChIP-Seq peaks were clustered near the consensus ERBS that we identified within the *MDM4* and *MDM2* genes (Supplementary Figure S5). Therefore, we conclude that ERα and FOXA1 directly access *MDM4* and *MDM2* chromatin, supporting a role for these factors in regulating *MDM4* and *MDM2* transcription.

Thus far, we have provided evidence that ERα positively regulates the expression of the *MDM4* and *MDM2* genes at the mRNA level (Figure 2B), which translates to the protein level (Figure 2A). We next used immunofluorescent microscopy to visualize the effect of loss of ERα on the subcellular localization and expression of MDM4 and MDM2 protein. Immunofluorescent staining of ERα in MCF7 cells revealed that the receptor was predominantly nuclear (Figure 2C, red fluorescence). Colabeling of MDM4 (green fluorescence) and ERα (red fluorescence) in the same cells demonstrated that MDM4 and ERα were both localized in the nuclear compartment (Figure 2C). Similar results were observed for MDM2 and ERα (Figure 2D). We next used ERα siRNA to transiently deplete ERα. Under this condition, nuclear MDM4 immunofluorescent signal was significantly reduced by approximately 50% (Figure 2C; green fluorescence), as was MDM2 signal (Figure 2D; green fluorescence). Due to the accessibility of many commonly-used, commercially-available MDM2 antibodies, we went on to validate our MDM2 immunofluorescence data with two additional MDM2 antibodies, both of which showed similar results (Supplementary Figure S2, panel B). We also verified that the immunofluorescent signals of our MDM4 and MDM2 antibodies were specific by demonstrating that siRNA-mediated knockdown of MDM4 and MDM2 could markedly reduce the immunofluorescent staining of each protein, respectively (Supplementary Figure S2, panels C and D).

**Figure 3:**
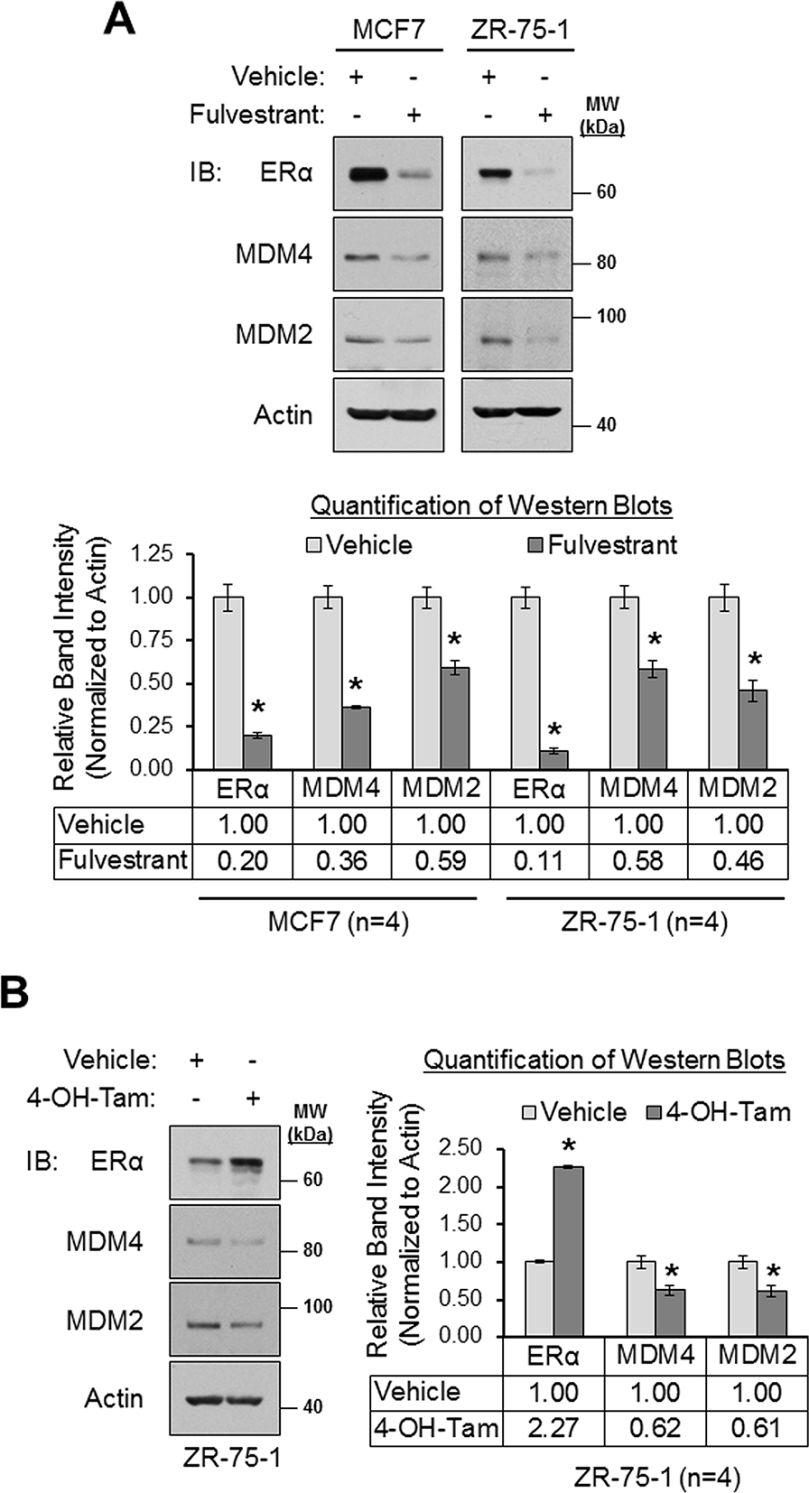
Pharmacologic inhibition of ERα results in the downregulation MDM4 and MDM2 in breast cancer cells (**A**) Western blot analysis of MCF7 and ZR-75-1 cells that were treated with vehicle (ethanol) or fulvestrant (1 μM) for 24 h. Upper panel: representative western blots. Lower panel: quantification of western blots (*n* = 4). (**B**) Western blot analysis of ZR-75-1 cells that were treated with vehicle (ethanol) or 4-hydroxytamoxifen (1 μM) for 24 h. Left panel: representative western blot. Right panel: quantification of western blots (*n* = 4). Error bars = SEM of biological replicates. *p < 0.05, unpaired *t*-test. 4-OH-Tam = 4-hydroxytamoxifen. IB = immunoblot. kDa = kilodaltons. MW = molecular weight.

To complement our ERα siRNA experiments, we next tested if MDM4 and MDM2 expression could be modulated pharmacologically with the clinically-relevant ERα antagonists, fulvestrant and 4-hydroxytamoxifen. Fulvestrant is a pure anti-estrogen that inhibits ERα function and downregulates the receptor [87–89]. 4-hydroxytamoxifen is a selective estrogen receptor modulator that inhibits ERα function but upregulates the receptor [90–92]. We observed that fulvestrant downregulated MDM4 and MDM2 expression in MCF7 cells by 64% and 41%, respectively, and in ZR-75-1 cells by 42% and 54%, respectively (Figure 3A). Similar to fulvestrant, 4-hydroxytamoxifen also downregulated both MDM4 and MDM2 expression by approximately 40% (Figure 3B). Collectively, the data presented thus far establish ERα as a positive regulator of MDM4 and MDM2 expression. Furthermore, we conclude that two ERα antagonists with different mechanisms of action, fulvestrant and 4-hydroxytamoxifen, can be used to inhibit MDM4 and MDM2 expression.

### ERα regulates p53 expression, but p53 is dispensable for ERα-mediated regulation of MDM4 and MDM2

**Figure 4:**
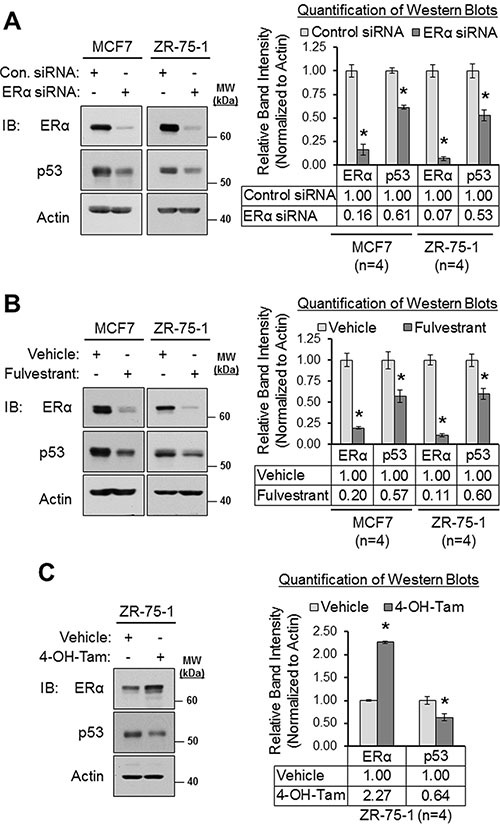
Loss of and/or inhibition of ERα downregulates p53 expression (**A**) Western blot analysis of MCF7 and ZR-75-1 cells that were transfected with control siRNA or ERα siRNA for 22 h. Left panel: representative western blot. Right panel: quantification of western blots (*n* = 4). (**B**) Western blot analysis of MCF7 and ZR-75-1 cells that were treated with vehicle (ethanol) or fulvestrant (1 μM) for 24 h. Left panel: representative western blot. Right panel: quantification of western blots (*n* = 4). (**C**) Western blot analysis of ZR-75-1 cells that were treated with vehicle (ethanol) or 4-hydroxytamoxifen (1 μM) for 24 h. Left panel: representative western blot. Right panel: quantification of western blots (*n* = 4). Error bars = SEM of biological replicates. *p < 0.05, unpaired *t*-test. 4-OH-Tam = 4-hydroxytamoxifen. Con. = control. IB = immunoblot. kDa = kilodaltons. MW = molecular weight.

Our results from Figures 2 and 3 revealed that loss of ERα signaling resulted in the downregulation of MDM4 and MDM2. Since MDM4 and MDM2 cooperate to negatively regulate p53 expression by promoting the polyubiquitination and proteasomal degradation of p53 [16, 20, 30], we tested if the downregulation of MDM4 and MDM2 that we observed in response to loss of ERα signaling would result in a subsequent increase in p53 expression. Surprisingly, we observed that conditions which downregulated MDM2 and MDM4 (i.e. ERα siRNA, fulvestrant and 4-hydroxytamoxifen; Figures 2 and 3) did not upregulate p53. Rather, they downregulated p53. As shown in Figure 4, ERα siRNA decreased p53 expression by approximately 40% in MCF7 cells and by approximately 50% in ZR-75-1 cells (panel A); fulvestrant downregulated p53 by approximately 40% in MCF7 and ZR-75-1 cells (panel B); and 4-hydroxytamoxifen reduced p53 expression by approximately 40% in ZR-75-1 cells (panel C). Therefore, we conclude that inhibition of ERα downregulates p53 expression along with MDM4 and MDM2. This is consistent with previous reports that demonstrated that ERα upregulates p53 expression [93-97] by binding directly to the *TP53/p53* promoter and transcriptionally activating the gene [93]. In addition, this finding also indicates that the downregulation of MDM4 and MDM2 in response to inhibition of ERα signaling is unlikely to have resulted in a significant loss of ubiquitination of p53, as an accumulation of p53 protein was not observed under these conditions.

**Figure 5:**
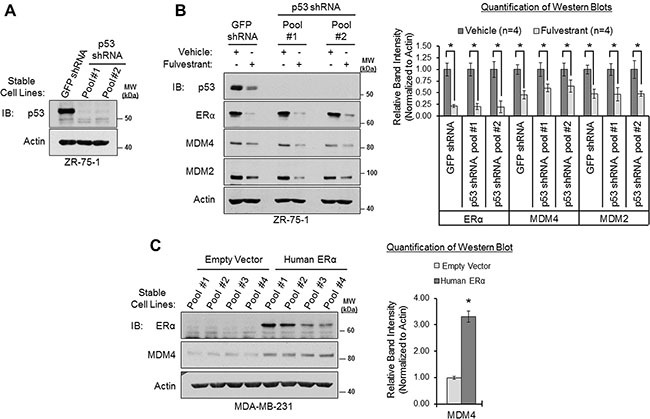
ERα modulates MDM4 expression via p53-independent mechanisms (**A**) Western blot analysis of ZR-75-1 cells that stably overexpress GFP shRNA or p53 shRNA (two different cell pools). (**B**) Western blot analysis of cells from panel A that were treated with vehicle (ethanol) or fulvestrant (1 μM) for 22 h. Left: representative western blot. Right: Quantification of western blots (*n* = 4). Error bars = SEM of biological replicates. (**C**) Western blot analysis of p53-mutant MDA-MB-231 cells that stably overexpress human ERα cDNA or an empty vector. Left: western blot. Right: quantification of western blot. Error bars = SEM of 4 different cell pools for each condition. *p < 0.05, unpaired *t*-test. IB = immunoblot. kDa = kilodaltons. MW = molecular weight.

We next tested if ERα's ability to positively regulate MDM4 and MDM2 expression was p53-dependent because we had observed that ERα positively regulates p53 expression (Figure 4), and it is known that p53 transcriptionally upregulates the *MDM4* and *MDM2* genes [31–37]. We generated two different pools of ZR-75-1 cells that stably expressed a p53-silencing shRNA construct. As shown in Figure 5A, the p53 shRNA efficiently knocked down p53 to nearly undetectable levels, when compared to control cells that expressed GFP shRNA. We then treated these shRNA-engineered cell lines with the ERα antagonist fulvestrant to downregulate and inhibit ERα, and we measured the expression of MDM4 and MDM2 by western blotting. We observed that fulvestrant downregulated MDM4 and MDM2 in both the p53-shRNA-expressing and the GFP-shRNA-expressing ZR-75-1 cells (Figure 5B). Notably, two different p53 shRNA-infected ZR-75-1 cell pools yielded similar findings, demonstrating the specificity of the effect (Figure 5B). In addition, comparable results were also observed in MCF7 cells, where we found that fulvestrant retained the ability to downregulate MDM4 and MDM2 in the absence of p53 in two different p53 shRNA-expressing MCF7 cell pools (Supplementary Figure S6). From these findings, we conclude that the ERα antagonist fulvestrant downregulates MDM4 and MDM2 via p53-independent mechanisms.

As a complementary approach, we also stably overexpressed ERα in the ERα-negative breast adenocarcinoma cell line, MDA-MB-231. This cell line expresses a hot spot point mutation (R280K) within the DNA-binding domain of p53, which significantly hinders p53′s ability to bind to cognate DNA response elements and, therefore, renders p53 transcriptionally inactive [98, 99]. A previous study has demonstrated that overexpression of ERα in this cell line results in the upregulation of MDM2 [68]. Therefore, we hypothesized that overexpression of ERα would also upregulate MDM4. Consistent with our hypothesis, we found that stable overexpression of human *ERα/ESR1* cDNA in four different pools of MDA-MB-231 cells led to a more than 3-fold upregulation of MDM4, as compared to four cell pools that stably expressed an empty vector (Figure 5C). This indicates that ERα can upregulate MDM4 in the absence of functional, transactivation-competent p53 protein. To summarize, the collective findings of Figure 5 and Supplementary Figure S6 indicate that ERα positively regulates MDM4 and MDM2 expression via p53-independent mechanisms. This is in agreement with our findings from Figure 1, where we observed that ERα expression correlated with *MDM4* and *MDM2* gene expression in primary breast carcinomas, independently of *TP53* mutational status.

### MDM4 and MDM2 negatively regulate ERα expression and form a protein complex with ERα

**Figure 6:**
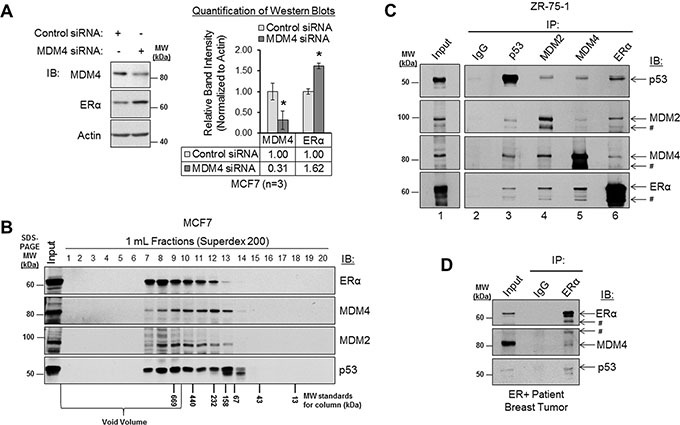
MDM4 and MDM2 negatively regulate ERα expression and form a protein complex with ERα (**A**) Western blot analysis of MCF7 cells that had been transfected with control siRNA or MDM4 siRNA for 48 h. Left panel: representative western blot. Right panel: quantification of western blots (*n* = 3). Error bars = SEM of biological replicates. *p < 0.05, unpaired *t*-test. (**B**) Western blot analysis of MCF7 lysates that were subjected to size exclusion column chromatography gel filtration. The fraction numbers are labeled above the blot. The molecular weight markers for column fractionation are labeled below the blot. The molecular weight markers for SDS-PAGE are labeled to the right of the blot. (**C**) Co-immunoprecipitation (co-IP) analysis of protein complexes in ZR-75-1 lysates. Immunoprecipitation (IP) was performed with normal IgG, p53 antibody, MDM2 antibody, MDM4 antibody or ERα antibody, as labeled above the blot. Immunoblot (IB) detection was performed using p53, MDM2, MDM4, or ERα antibodies, as labeled to the right of the blot. IgG is shown as a negative control. Interactions between p53-MDM2, p53-MDM4 and p53-ERα are shown as positive controls. (**D**) Co-IP analysis of protein complexes in lysate from a primary ERα-positive patient breast tumor sample. IP was performed with normal IgG or ERα antibody, as labeled above the blot. IB detection was performed using ERα, MDM4 or p53 antibodies, as labeled to the right of the blot. IgG is shown as a negative control. Interaction between p53-ERα is shown as a positive control. ^#^ = related isoform, protein fragment, or non-specific band. IB = immunoblot. IgG = immunoglobulin G. IP = immunoprecipitation. kDa = kilodaltons. MW = molecular weight.

We have presented evidence that ERα positively regulates MDM4 and MDM2 expression. However, a previous study has also demonstrated that MDM2 negatively regulates ERα expression [69]. Together, these findings suggest that a negative feedback loop exists between ERα and MDM2 at the expression level. We further hypothesized that a similar negative feedback loop may exist to opposingly regulate ERα and MDM4 expression. To test this hypothesis, we first confirmed that MDM2 negatively regulates ERα expression. Consistent with a previous report [69], western blot analysis of MCF7 cells that had been transiently transfected with MDM2 siRNA or non-silencing control siRNA revealed that loss of MDM2 resulted in an approximately 50% increase in ERα expression (Supplementary Figure S7, panel A). Similarly, siRNA-mediated knockdown of MDM4 also increased ERα expression by approximately 60% in MCF7 cells (Figure 6A). Therefore, not only does ERα positively regulate MDM4 and MDM2 expression (Figures 2 and 3), but MDM4 and MDM2 also negatively regulate ERα expression (Figure 6A and Supplementary Figure S7, panel A). This finding is indicative of the existence of negative feedback loops that regulate ERα and MDM4 expression and that similarly regulate ERα and MDM2 expression.

It has been proposed that MDM2 negatively regulates ERα expression by forming a protein complex with ERα, which either directly or indirectly facilitates the ubiquitination and proteasomal degradation of the receptor [69]. However, studies have demonstrated that MDM2 and MDM4 preferentially exist as a heterodimer [16, 100], and the MDM2/MDM4 heterodimeric complex is a more potent E3 ubiquitin ligase than the MDM2 homodimer [10, 16, 20, 30, 101]. Based on these lines of evidence, we hypothesized that MDM4, like MDM2, would be capable of forming a protein complex with ERα. Although protein complexes consisting of MDM2-ERα, MDM2-MDM4, MDM2-p53, MDM4-p53 and ERα-p53 have been identified [18, 21, 23, 27, 28, 41, 43, 66, 69, 102–108], complexes consisting of MDM4-ERα have never before been described. In addition, protein complexes comprised of endogenously-expressed MDM2 and ERα proteins have not been characterized in breast cancer cells.

Having already established that ERα, MDM4 and MDM2 are all localized in the nuclear compartment of breast cancer cells (Figure 2), we next used size exclusion column chromatography and co-immunoprecipitation (co-IP) assays to assess the ability of ERα to form a protein complex with MDM4, as compared to MDM2 and p53, which served as positive controls. Size exclusion column chromatographic analyses of MCF7 lysates revealed that a portion of ERα co-fractionated with MDM4, MDM2 and p53 in high molecular weight fractions (Figure 6B), supporting the potential for these proteins to form a complex with one another. We then analyzed endogenous protein complexes comprised of ERα and MDM4 by co-IP in ZR-75-1 cells (Figure 6C) and MCF7 cells (Supplementary Figure S7, panel B). Using MDM4 antibody (lane 5) or ERα antibody (lane 6) in reciprocal co-IP assays, we found that MDM4 was indeed in complex with ERα in both cell lines. As positive controls for previously identified protein complexes, we observed that p53 antibody (lane 3) co-immunoprecipitated MDM2, MDM4, and ERα. Also, MDM2, MDM4, and ERα antibodies (lanes 4, 5, and 6, respectively) co-immunoprecipitated p53 in reciprocal co-IP experiments. Likewise, interactions between endogenous MDM2 and MDM4 were also detected in co-IP assays when we used either MDM2 antibody (lane 4) or MDM4 antibody (lane 5) for immunoprecipitation, as were interactions between ERα and MDM2 when we used either ERα antibody (lane 6) or MDM2 antibody (lane 4) for immunoprecipitation. As a negative control, normal IgG (lane 2) failed to immunoprecipitate any of our proteins of interest.

To determine if protein complexes consisting of ERα and MDM4 were relevant *in vivo*, we conducted a co-IP experiment using lysate from an ERα-positive, treatment-naive, primary breast carcinoma patient tissue sample. Again, we observed that MDM4 co-immunoprecipitated with ERα (Figure 6D). The interaction between ERα and p53 is shown as a positive control. As a negative control, normal IgG failed to immunoprecipitate ERα, MDM4, and p53. Therefore, we conclude that ERα is found in complex with MDM4 in human breast cancer cell lines and in patient breast tumors. This is the first report to demonstrate that ERα and MDM4 exist in a protein complex with one another, and it is also the first report to demonstrate that endogenous ERα and MDM2 similarly complex with one another in breast cancer cells.

**Figure 7:**
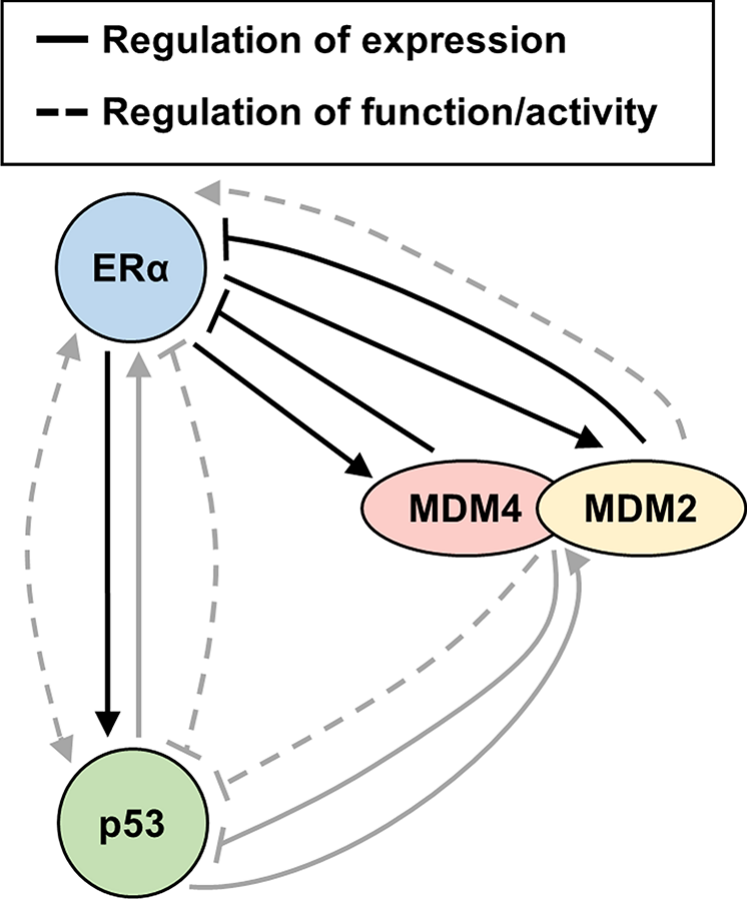
Summary diagram Schematic diagram of feedback loops and signaling crosstalk amongst MDM4, MDM2, ERα and p53 that govern their expression and function. Findings from the present report are indicated with black arrows. Findings from prior studies are indicated with grey arrows. The present study demonstrates that ERα positively regulates MDM2 and MDM4 expression, while MDM2 and MDM4 negatively regulate ERα expression, in agreement with a previous report that also demonstrated that MDM2 negatively regulates ERα expression [69]. In addition, it is known from prior reports that MDM2 enhances the transactivation function of ERα [41, 43]. In prior studies, p53 and ERα have been demonstrated to mutually support one another's expression, in agreement with our finding from the present study where we observed that ERα positively regulates p53 expression; however, it is known that p53 and ERα inhibit one another's ability to regulate the expression of some genes and cooperate with one another to mutually upregulate other genes [93–97, 102, 105, 108, 114, 141–145]. MDM2 and MDM4 negatively regulate p53 expression via ubiquitination and also repress the transactivation function of p53; yet, p53 transcriptionally induces MDM2 and MDM4 expression [1–4, 31–37]. Additional levels of regulation that are not included in the diagram include the ability of MDM4 to promote p53-mediated transcriptional upregulation of MDM2 [130], the ability of the MDM2/MDM4 heterodimer to negatively regulate its own expression via auto-ubiquitination [10, 20, 30, 101, 146], and the ability of MDM2 and MDM4 to enhance p53 translation under conditions of cell stress [147, 148]. Furthermore, we have herein demonstrated that all four of the illustrated proteins form endogenous protein complexes with one another in breast cancer cells.

To summarize, our collective findings describe a negative feedback loop, wherein ERα upregulates MDM4 and MDM2 expression, and in turn, MDM4 and MDM2 downregulate ERα expression. This builds upon the complex regulatory loops that have previously been described for p53, ERα, MDM4, and MDM2 (summarized in Figure 7) and link ERα to MDM4 for the first time. In addition, we have identified MDM4 as a novel ERα-interacting protein, and we confirm prior reports that MDM2 and p53 also form a protein complex with ERα. These findings point to the existence of important signaling crosstalk amongst ERα, MDM4, MDM2 and p53 in human breast cancer.

## DISCUSSION

Previous studies have demonstrated that MDM2 is overexpressed in human breast cancer and that ERα is coexpressed with MDM2 and regulates *MDM2* gene expression [38, 47, 49–54, 56, 61–68]. The MDM2 homolog, MDM4, is also overexpressed in human breast cancer [49, 53, 55, 70]. However, the mechanisms underlying its overexpression remained to be elucidated. In the present work, we report that ERα expression correlates not only with *MDM2* gene expression in primary breast carcinoma samples but also with *MDM4* expression. In addition, we demonstrate that tumors of luminal A and luminal B molecular subtypes have the highest expression of *MDM2* and *MDM4* mRNA, in agreement with a prior study that observed that MDM4 protein expression is similarly elevated in luminal A/B breast tumors [55]. We also provide evidence that ERα mediates the p53-independent upregulation of MDM4 in human breast cancer cell lines, thereby providing an explanation for why MDM4 is overexpressed in ERα-positive luminal breast cancer and also identifying a novel p53-independent mechanism of regulating MDM4 expression. Other groups have also reported additional factors that similarly regulate either MDM2 or MDM4 expression independently of p53, such as MAPK, which regulates MDM4 expression, and NF-KB, TGFβ/SMAD and PTEN, which regulate MDM2 expression [73, 109–112]. Based on our observations from ChIP datamining studies, we postulate that ERα-dependent overexpression of MDM4 and MDM2 in human breast cancer is mediated, at least in part, by the ability of ERα to directly bind to and transcriptionally upregulate the *MDM4* and *MDM2* genes. We also observed that ERα positively regulates p53 expression; a finding that is supported by a recent study that reported that ERα directly regulates *TP53/p53* gene transcription [93]. Together, these findings indicate that ERα supports the expression of the entire MDM4-MDM2-p53 signaling axis. We have herein provided evidence that ERα-targeting therapies block the ability of ERα to modulate MDM4 and MDM2 expression. Therefore, as enthusiasm for the preclinical development of novel MDM4/MDM2 inhibitors builds (reviewed in [3]), we propose that these future agents may show utility in combination with hormonal therapies for the treatment of MDM4/MDM2-driven breast cancers.

Why does ERα upregulate MDM4 and MDM2 expression, and what is the physiological relevance of such regulation? Since MDM4 and MDM2 play well-characterized proliferative and prosurvival roles in breast cancer models [38–44, 55, 70–72], ERα-dependent upregulation of MDM4 and MDM2 surely enhances these processes. In addition, ERα may modulate MDM4 and MDM2 expression for the purpose of inhibiting p53 function, despite upregulating p53 expression. Surprisingly, we found that the downregulation of MDM4 and MDM2 in response to loss of ERα signaling did not result in an increase in p53 expression, indicating that the p53 degradation pathway was unlikely to have been compromised. This points to the existence of MDM4/MDM2-independent p53 degradation pathways in breast cancer cells. Indeed, many other E3 and E4 ubiquitin ligases have been implicated in p53 ubiquitination (reviewed in [113]). Aside from ubiquitinating p53, MDM4 and MDM2 also inhibit p53′s transactivation domain. We propose that ERα-dependent modulation of MDM4 and MDM2 expression might not affect p53 ubiquitination but may negatively impact the ability of p53 to regulate gene transcription. In support of this hypothesis, in our previous studies, we have demonstrated that loss of ERα results in the upregulation of p53-target genes and that ERα antagonizes p53′s transactivation function in breast cancer cells [102, 103]. In these reports, we discovered that ERα-dependent inhibition of p53-target gene expression was mediated, at least in part, by the ability of ERα to form a protein complex with p53 and to recruit a co-repressor complex to p53 response elements [102, 103]. We now demonstrate in the present study that ERα not only forms a protein complex with p53, but also with MDM4 and MDM2; and furthermore, ERα upregulates the expression of MDM4 and MDM2—two proteins that can potently repress p53′s transactivation domain [18, 23, 26-28]. Therefore, future studies should determine if ERα partly antagonizes the ability of p53 to regulate target gene transcription via MDM4/MDM2-dependent mechanisms. Such a finding would likely have a global impact on p53 gene expression signatures in ERα-positive cells, as we have herein demonstrated that MDM4 and MDM2 are highly expressed in ERα-positive tumors, and Bailey et al. have demonstrated in a genome-wide breast cancer study that ERα antagonizes the expression of approximately 150 putative p53-regulated transcripts [114].

We also studied crosstalk between ERα, MDM2, and MDM4 in terms of protein-protein interactions. Despite protein-protein interactions having been described previously for MDM2-ERα, MDM2-MDM4, MDM2-p53, MDM4-p53 and ERα-p53 [18, 21, 23, 27, 28, 41, 43, 66, 69, 102–108], our study is the first to describe an interaction between ERα and MDM4 and to identify protein complexes comprised of endogenously-expressed ERα and MDM2 in breast cancer cells. Whether or not ERα binds directly to MDM4, or if MDM2 and/or p53 facilitate their interaction remains to be elucidated in future studies. However, it is conceivable that the four proteins exist in a single complex with one another and may function together to regulate several biological processes. At present, few MDM4-interacting proteins have been discovered, highlighting the novelty of our work. However, MDM2 has been reported to interact with a great number of proteins, including ATF3, AR, DYRK2, GR, HIPK2, hnRNP-K, IGF-1R, JMY, Nbs1, Notch4, Numb, TAB1, and various ribosomal proteins, to name only a few [2, 115–117]. It is likely that the protein interaction between ERα and MDM2/MDM4 and the ability of ERα to upregulate MDM2/MDM4 expression either directly or indirectly influences the abilities of MDM2/MDM4 to bind to and to regulate the function or expression of other proteins; therefore, ERα may contribute to breast oncogenesis by impinging upon MDM2/MDM4-dependent cellular processes that had not been speculated previously to be associated with ERα activity.

Additionally, the ERα-MDM4-MDM2 protein complex might also play important roles in positively regulating the already characterized protumorigenic functions of ERα, such as the ability of ERα to transactivate target genes. Two groups of investigators have shown that MDM2 enhances ERα's transactivation function [41, 43] and that MDM2 may play a general role in mediating transcriptional crosstalk between ERα and steroid receptor coactivator (SRC) proteins [41]. Furthermore, MDM2 is recruited with ERα to ERα-target genes in chromatin immunoprecipitation assays [89, 118, 119], and loss of MDM2 blocks the ability of ERα to transcriptionally upregulate its targets [41]. It has long been known that transcriptional activation of ERα-target genes by 17β-estradiol is linked to the subsequent proteasomal degradation of the receptor [87, 120–124]; therefore, it is possible that an additional function of the ERα-MDM4-MDM2 protein complex might be to regulate the ubiquitination of activated-ERα in concert with gene transcription. Since MDM4 and MDM2 preferentially exist as a heterodimer, and since heterodimerization is required for their potent E3 ligase activity [10, 16, 20, 21, 30, 100], we speculate that both MDM4 and MDM2 would be required to ubiquitinate ERα. In support of this hypothesis, we have demonstrated that both MDM4 and MDM2 form a protein complex with ERα and both proteins negatively regulate ERα expression. While MDM2 has been implicated in either the direct or indirect ubiquitination of many proteins—including the nuclear receptors ERα, ERβ, and androgen receptor [69, 125–127]—few studies have assessed the ability of MDM4 to ubiquitinate substrate proteins, other than its MDM2-dependent roles in auto-ubiquitination and the ubiquitination of p53 and MDM2 [10, 16, 20, 30, 101]. Additional investigation is presently underway to assess the role of MDM4 in regulating both the activity and proteasomal degradation of nuclear receptors via MDM2-dependent processes.

In conclusion, we have herein demonstrated that ERα expression associates with *MDM4* and *MDM2* gene expression in primary breast invasive carcinoma samples. Our *in vitro* analyses of human breast cancer cells reveal that ERα positively regulates MDM4 and MDM2 expression via p53-independent mechanisms, while MDM4 and MDM2 negatively regulate ERα expression. In addition, ERα forms a protein complex with MDM4, MDM2 and p53, suggesting direct signaling crosstalk amongst these four proteins. Future studies are needed to ascertain the intricate mechanisms which govern signaling crosstalk between ERα, MDM4 and MDM2, and to delineate the physiological relevance of such crosstalk in terms of p53 and ERα biology, breast tumorigenesis, and therapeutic response.

## MATERIALS AND METHODS

### Ethics statement

For the co-IP assay, de-identified patient breast tumor tissue was obtained from the Roswell Park Cancer Institute (RPCI) Pathology Resource Network. Investigation has been conducted in accordance with ethical standards of, the Declaration of Helsinki and has been approved by the authors' institutional review board. For the analysis of the Cancer Genome Atlas (TCGA) breast invasive carcinoma (BRCA) cohort, de-identified patient data from the public domain were used in accordance with TCGA data portal user guidelines.

### Gene expression analysis of the cancer genome atlas invasive breast carcinoma cohort

The Cancer Genome Atlas invasive breast carcinoma (TCGA BRCA) dataset is available from TCGA data portal (https://tcga-data.nci.nih.gov/tcga/). We downloaded the following data (as described by TCGA Network [75]) for TCGA BRCA cohort: de-identified clinical data of 949 patients, somatic mutation data of 825 patients, intrinsic molecular subtype assignment of 522 patients, and normalized RNA-Seq gene expression data of 778 patients using RNA-Seq by Expectation Maximization (RSEM) [128].

Patients with available RNA expression data, available clinical ERα status, available *TP53*, *MDM2*, and *MDM4* somatic mutation/amplification data, and available intrinsic molecular subtype assignment data were used to study *MDM2* and *MDM4* gene expression. 38 patients with *MDM2* amplification and 109 patients with *MDM4* amplification (according to [75]) were excluded from the analyses of *MDM2* and *MDM4* gene expression, respectively, to study effects on gene expression that were independent of gene amplification. The patient cohort was classified into five different intrinsic molecular subtypes (luminal A, luminal B, HER-enriched, basal-like and normal-like) using RNA expression of the PAM50 50-gene subtype predictor [76], according to [75]. For gene expression analysis between two groups, the *t*-test was applied. For gene expression analysis amongst more than two groups, an analysis of variance (ANOVA) was applied. The Pearson correlation test was applied to assess correlations between *MDM2/MDM4* and *ESR1/ERα* mRNA expression. All statistical tests were carried out using R statistical software and *p*-values of less than 0.05 were considered to be statistically significant.

### Cell culture and reagents

The human breast cancer cell lines MCF7, ZR-75-1 and MDA-MB-231 (ATCC) were maintained in Dulbecco's Modified Eagle's Medium (DMEM; Mediatech) supplemented with 10% fetal bovine serum (FBS; Life Technologies) at 37°C, under a humidified atmosphere of 5% carbon dioxide. 4-hydroxytamoxifen (Sigma) and fulvestrant (Tocris Bioscience) were solubilized in ethanol, and final ethanol concentrations in the media of treated cells were less than 0.01%. The ERα expression plasmid was kindly provided by Dr. Carolyn Smith (Baylor College of Medicine, Houston, TX).

### Small interfering RNA (siRNA)

siRNA transfections were performed as previously described [129]. In brief, siRNA at a final concentration of 50 nM was transfected into cells using Lipofectamine 2000 (Life Technologies), according to the manufacturer's instructions. Cells were harvested post-transfection at the times indicated in the figure legends. The ERα siRNA sequences were as follows: sequence #1 (Ambion, siRNA ID # s4824) and sequence #2 (Dharmacon, catalog # M-003401-04-0020). The MDM2 siRNA was a 1:1 mix of two different sequences: sequence #1 (Qiagen, catalog # SI02653392) and sequence #2 (Ambion, siRNA ID # HSS142909). The MDM4 siRNA was a 1:1 mix of two different sequences: sequence #1 (Qiagen, catalog # SI00037163) and sequence #2 (Ambion, custom sequence; sense 5′-GGA UAU UCC AAG UCA AGA CUU-3′; antisense 5′-GUC UUG ACU UGG AAU AUC CAU-3′; as described in [130]). The non-silencing control siRNA was from Ambion (catalog # 4390843).

### Sodium dodecyl sulfate polyacrylamide gel electrophoresis (SDS-PAGE) and western blotting

MCF7, ZR-75-1 and MDA-MB-231 cells were harvested in phosphate buffered saline (PBS; Mediatech) and pelleted by centrifugation at 1,000 rpm. Cell pellets were then lysed in ice cold lysis buffer [20 mM Tris, pH 8.0; 150 mM NaCl; 5 mM MgCl_2_; 0.5% NP40; 2 mM PMSF; 1X EDTA-free Complete protease inhibitor cocktail (Roche)] for 30 minutes with intermittent gentle vortexing. Lysates were centrifuged at 10,000 rpm for 10 min at 4°C to remove debris. Protein concentrations were assessed by the Bradford method using Protein Assay Dye Reagent Concentrate (Bio-Rad), a BSA standard curve, and a BioTek Synergy 2 plate reader with Gen5 v1.11.5 software. Protein lysates were mixed with 4X Laemmli buffer [200 mM Tris, pH 6.8; 8% SDS; 40% glycerol; 20% 2-mercaptoethanol; 0.4% bromophenol blue] at ratios that yielded a 1X final concentration of Laemmli buffer, boiled at 100°C for 10 min, and subjected to 8% SDS-PAGE. Proteins were transferred to PVDF membranes, blocked for 45 min with 5% non-fat milk dissolved in Tris-buffered saline-tween (TBST) [10 mM Tris, pH 8.0; 150 mM NaCl; 0.05% Tween-20], and incubated with primary antibody diluted in 0.5% non-fat milk-TBST overnight at 4°C with gentle rocking. The following day, membranes were washed 3 times with TBST, incubated with horseradish peroxidase-conjugated secondary antibody diluted in 0.5% non-fat milk-TBST for 3 h at room temperature with gentle rocking, and then washed 3 times with TBST. Bands were visualized using the chemiluminescent method with ECL Western Blotting Substrate (Pierce), and films were developed using a Kodak X-Omat 200A Processor. Films were scanned using a Bio-Rad SG-800 calibrated densitometer and Quantity One v4.6.7 software. Bands were quantified using Image J software, and relative band intensity was calculated as relative to control, after having been normalized to β-Actin. Error bars are representative of standard error of the means of either triplicate or quadruplicate biological replicates, as indicated in the figure legends. Statistically significant differences in the means were calculated using unpaired *t*-tests.

Primary antibodies for western blotting were as follows: p53 (DO-1, Santa Cruz, catalog # sc-126) used at 1:1,000; ERα (HC-20, Santa Cruz, catalog # sc-543) used at 1:10,000; MDM2 (IF2, EMD Millipore, catalog # MABE340) used at 3:1,000; MDM4 (Bethyl Labs, catalog # A300-287A) used at 1:10,000; c-Myc (Y69, Abcam, catalog # ab32072) used at 1:2,000; and β-Actin (AC-15, Sigma, catalog # A5441) used at 1:10,000.

Secondary antibodies for traditional western blotting were as follows: goat anti-rabbit IgG HRP-conjugate (EMD Millipore, catalog #12-348) used at 1:10,000 and goat anti-mouse IgG HRP-conjugate (EMD Millipore, catalog # 12-349) used at 1:10,000. For western blotting following co-immunoprecipitation, light chain-specific secondary antibodies were used to prevent the detection of the antibody heavy chain. Goat anti-mouse light chain specific IgG-HRP conjugate was used at 1:10,000, and mouse anti-rabbit light chain specific IgG-HRP conjugate was used at 1:20,000 (Jackson Immuno Research, catalog # 211-032-171 and # 115-035-174, respectively).

### Quantitative real time polymerase chain reaction (qPCR)

Total RNA was isolated using Trizol Reagent (Life Technologies) and then purified by sodium acetate ethanol precipitation. RNA was quantified using a Nanodrop 8000 spectrophotometer. 1 μg of total RNA was subjected to DNase I (Life Technologies; amplification grade) digestion in a 10 μL reaction, according to the manufacturer's instructions. DNase I-digested RNA was then reverse transcribed in a 20 μL reaction using the iScript cDNA Synthesis Kit (Bio-Rad) and a Bio-Rad C1000 Touch thermocycler, according to the cycling parameters described in the manufacturer's instructions. cDNA was diluted 4-fold with molecular grade water prior to qPCR. qPCR was carried out in an ABI 7300 Real Time PCR System with 7300 System SDS Software (Applied Biosystems) in a 10 μL reaction containing iTaq Universal SYBR Green Supermix (Bio-Rad), 1.5 μL of diluted cDNA, and 500 nM of specific primers. The cycling conditions were as follows: 50°C for 15s (1 cycle); 95°C for 10 min (1 cycle); 95°C for 15 s, followed by 60°C for 45 s (35 cycles). The detection of a single amplicon was verified using a dissociation curve. Normalized, relative mRNA levels were calculated according to the ΔΔ*C*_q_ method, using endogenous *ACTB* as a reference gene for normalization. Error bars are representative of standard error of the means of quadruplicate biological replicates, as indicated in the figure legends. Statistically significant differences in the means were calculated using unpaired *t*-tests.

Primer efficiencies were calculated from a dilution curve and determined to be within the acceptable range of 90–110% efficiency [131]. Primers were designed using Primer 3 software [132, 133] or taken from published sources, as indicated below. Primers were verified to amplify a single PCR product by traditional agarose gel electrophoresis. The following primer sets were used for qPCR: *ACTB* (RefSeq NM_001101.3), 5′-ATG GGT CAG AAG GAT TCC TAT-3′ and 5′-AAG GTC TCA AAC ATG ATC TGG G-3′, as described in [102]; *ESR1* (RefSeq NM_000125.3), 5′-GCA GTG TGC AAT GAC TAT G-3′ and 5′-CGT TAT GTC CTT GAA TAC TTC-3′; *MDM4* (RefSeq NM_002393.4), 5′-ATC TGA CAG TGC TTG CAG GA-3′ and 5′-GCT GCA TGC AAA ATC TTC AA-3′; and *MDM2* (RefSeq NM_002392.5), 5′-GGT CGA CCT AAA AAT GGT TGC A-3′ and 5′-GGG CAG GGC TTA TTC CTT TTC-3′, as described in [134].

### Chromatin Immunoprecipitation-Sequencing (ChIP-Seq) and ChIP-on-Chip (ChIP-Chip) cistromic data mining

For ERα ChIP-Seq and ChIP-Chip datasets, normalized ERα ChIP bed files and wig files were downloaded from the Nuclear Receptor Cistrome Project database (http://cistrome.org/NR_Cistrome/Cistrome.html; [80]) and the ENCODE Project database (https://www.encodeproject.org; [81]). The following datasets were from Nuclear Receptor Cistrome: Antoni Hurtado dataset, unpublished data; Edison Liu dataset, (Gene Expression Omnibus (GEO) Accession: GSE23701) [85]; Hendrik Stunnenberg dataset, (GEO Accession: GSE14664) [135]; Duncan Odom dataset, (Array Express Accession: E-TABM-828) [136]; Arul Chinnaiyan dataset, (GEO Accession: GSE19013) [137]. Myles Brown dataset (http://research.dfci.harvard.edu/brownlab/datasets/index.php?dir=ER_whole_human_genome) [84]; and Kevin White dataset, (GEO Accession: GSE15244) [138]. The following dataset was from ENCODE: Richard Meyers/ENCODE Consortium dataset, (GEO accession: GSE32465) [81]. Bed and wig files were viewed using Integrated Genomics Viewer (IGV) software [139]. Data are displayed in hg19 alignment format.

Normalized FOXA1 ChIP bed files were downloaded from the from the Jason Carroll lab website (http://www.carroll-lab.org.uk/data) and are described in [86]. Bed files were converted from hg18 genomic coordinates to hg19 genomic coordinates using the UCSC Batch Coordinate Conversion Lift Over Tool software (https://genome.ucsc.edu/cgi-bin/hgLiftOver), described in [140]. Bed files were viewed using IGV.

### Immunofluorescent (IF) microscopy

MCF7 cells were seeded onto sterile glass coverslips in 6-well plates at a density of 0.2 million cells per well. The following day, cells were transfected with siRNA. After the times indicated in the figure legends, cells were rinsed twice with PBS (Mediatech) and then fixed with 2 mL of fixation buffer [4% paraformaldehyde; PBS, pH 7.4] for 15 min at room temperature with gentle rocking. Coverslips were then rinsed 4 times with PBS prior to incubation with 2 mL of permeabilization buffer [0.5% Triton X-100; PBS, pH 7.4] for 10 minutes at room temperature with gentle rocking. Coverslips were rinsed 3 times with PBS and then blocked with blocking buffer [3% non-fat milk; PBS, pH 7.4] for 1 h at room temperature with gentle rocking. Coverslips were rinsed once with PBS and then incubated with primary antibodies diluted in 1% non-fat milk-PBS for 3.5 h at room temperature. Coverslips were rinsed 3 times with PBS and then incubated with secondary antibodies diluted in 0.5% non-fat milk-PBS for 1.5 h at room temperature. Coverslips were rinsed once with PBS and then incubated with a 1 mg/mL solution of 4′,6-diamidino-2-phenylindole (DAPI) diluted in PBS for 15 min at room temperature. Coverslips were rinsed 2 times with PBS and then 1 time with water, prior to being mounted onto slides with ProLong Gold antifade medium (Life Technologies) and CoverGrip Coverslip Sealant (Biotium).

Cells were visualized with a Zeiss epifluorescence microscope and 100X Plan-Neofluar, 63X Plan-Apochromat, and 40X Plan-Neofluar objective lenses. Images were obtained with a digital camera and Image-Pro Plus v4.5.1.29 or Spot Advanced v5.2 software.

Primary antibodies for IF were as follows: ERα HC-20 antibody (Santa Cruz, catalog # sc-543) at 1:200; MDM2 SMP14 antibody (Santa Cruz, catalog # sc-965) at 1:100; MDM2 4B11 antibody (EMD Millipore, catalog # OP143) at 1:25; MDM2 IF2 antibody (EMD Millipore, catalog # MABE340) at 1:50; and MDM4 C86 antibody (Millipore catalog # 04-1555) at 1:25.

Secondary antibodies for IF were as follows: Alexa Fluor 488 Goat anti-Mouse IgG (H+L) (Invitrogen Molecular Probes, catalog # A11001) and Alexa Fluor 595 Goat anti-Rabbit IgG (H+L) (Invitrogen Molecular Probes, catalog # A11012), both used at 1:500.

### Generation of stable cell lines

To generate p53 knockdown cells and corresponding control cells, MCF7 and ZR-75-1 cells were infected with lentiviral particles expressing pLKO.1-p53 shRNA or pLKO.1-GFP shRNA (kind gifts from Dr. Xinjiang Wang, Roswell Park Cancer Institute, described in [134]), followed by selection with puromycin for one week at 2 μg/mL. The appropriate puromycin concentration was determined from a kill curve. Two different p53 shRNA pools were generated for each cell line.

To generate ERα-overexpressing cells, MDA-MB-231 cells were transfected with 6 μg of PCR3.1 plasmid (Invitrogen), encoding either human ERα cDNA (described in [103]) or no insert. Cells were selected with 800 μg/mL of G418 sulfate (Mediatech) for 3 weeks and were maintained in 400 μg/mL of G418 sulfate. The appropriate G418 sulfate concentration was determined from a kill curve. Four different ERα-overexpressing pools and four different empty vector pools were generated.

### Size exclusion column chromatography

MCF7 cells were lysed in ice cold gel filtration lysis buffer [50 mM HEPES, pH 7.4; 150 mM NaCl; 0.5% NP-40; 1X EDTA-free Complete Protease Inhibitor Cocktail (Roche)] at 4°C with gentle rotation for 30 min. Lysates were then centrifuged twice at 15,000 rpm for 15 min to remove debris. 2.5 mg of lysate was then fractionated by size-exclusion chromatography at 4°C, using a Superdex 200 10/300 GL column, an AKTA Purifier system and Unicorn 5.1 software (GE Healthcare). Protein complexes were eluted using column buffer [25 mM HEPES, pH 7.4; 150 mM NaCl], and 24 fractions of 1 mL each were collected. Equal volumes of each of the different fractions were then analyzed by western blotting. The column was calibrated with the following molecular weight standards: Blue Dextran 2000, thyroglobulin, ferritin, catalase, aldolase, bovine serum albumin, ovalbumin, and ribonuclease A (GE Healthcare). Based on the Blue Dextran 2000 elution profile, the void volume was determined to be 9 mL, in agreement with the manufacturer's predicted void volume.

### Co-immunoprecipitation (Co-IP)

ZR-75-1 and MCF7 cells were harvested by scraping in PBS (Mediatech) and pelleted by centrifugation at 1,000 rpm. Cell pellets were lysed in ice cold lysis buffer [20 mM Tris, pH 8.0; 150 mM NaCl; 5 mM MgCl_2_; 0.5% NP40; 1X EDTA-free Complete protease inhibitor cocktail (Roche)] for 30 minutes with intermittent gentle vortexing. Lysates were centrifuged at 15,000 rpm for 10 min at 4°C and then pre-cleared with protein G agarose (Life Technologies) for 1 h at 4°C with gentle rotation. 2.5 mg of precleared lysate was then incubated overnight with 6 μg of antibody at 4°C with gentle rotation. The following day, immunogen-antibody complexes were captured by incubation with protein G agarose for 3 h at 4°C with gentle rotation. The protein G agarose was then washed 4 times with wash buffer [20 mM Tris, pH 8.0; 150 mM NaCl; 5 mM MgCl_2_; 1X EDTA-free Complete protease inhibitor cocktail (Roche)], the agarose pellets were collected, and residual wash buffer was removed. Protein complexes were eluted in 2X Laemmli buffer [100 mM Tris, pH 6.8; 4% SDS; 20% glycerol; 10% 2-mercaptoethanol; 0.2% bromophenol blue], boiled at 100°C for 10 min and then analyzed by 7% SDS-PAGE, followed by western blotting, as described above.

Co-IP of human breast tumor lysate was conducted with the following modification; 3 mg of fresh, frozen breast carcinoma tissue from a treatment-naive patient was pulverized using a dounce homogenizer prior to lysis.

IP antibodies were as follows: Normal mouse and normal rabbit IgG (EMD Millipore, catalog # 12-371 and # 12-370); p53 antibodies as a 1:1:1 mix of DO-1 (Santa Cruz, catalog # sc-126), FL-393 (Santa Cruz, catalog # sc-6243) and PAb421 (EMD Millipore, catalog # OP03L); ERα antibodies as a 1:1 mix of HC-20 (Santa Cruz, catalog # sc-543) and D-12 (Santa Cruz, catalog # sc-8005); MDM2 antibodies as a 1:1:1 ratio of SMP14 (Santa Cruz, catalog # sc-965), N-20 (Santa Cruz, catalog # sc-913) and 4B11 (EMD Millipore, catalog # OP143); and MDM4 antibody (Bethyl Labs, catalog # A300-287A).

## SUPPLEMENTARY MATERIALS FIGURES AND TABLES


