# Supplementary material for: Estrogen receptor alpha (ERα/ESR1) mediates the p53-independent overexpression of MDM4/MDMX and MDM2 in human breast cancer
Source: Oncotarget. 2016 Feb 20;7(13):16049–69. doi: 10.18632/oncotarget.7533 (PMC4941297; doi:10.18632/oncotarget.7533)
Supplement: Supplementary file 1 [file oncotarget-07-16049-s001.pdf]

Estrogen receptor alpha (ERα/ESR1) mediates the p53-independent overexpression of MDM4/MDMX and MDM2 in human breast cancer

Supplementary Materials

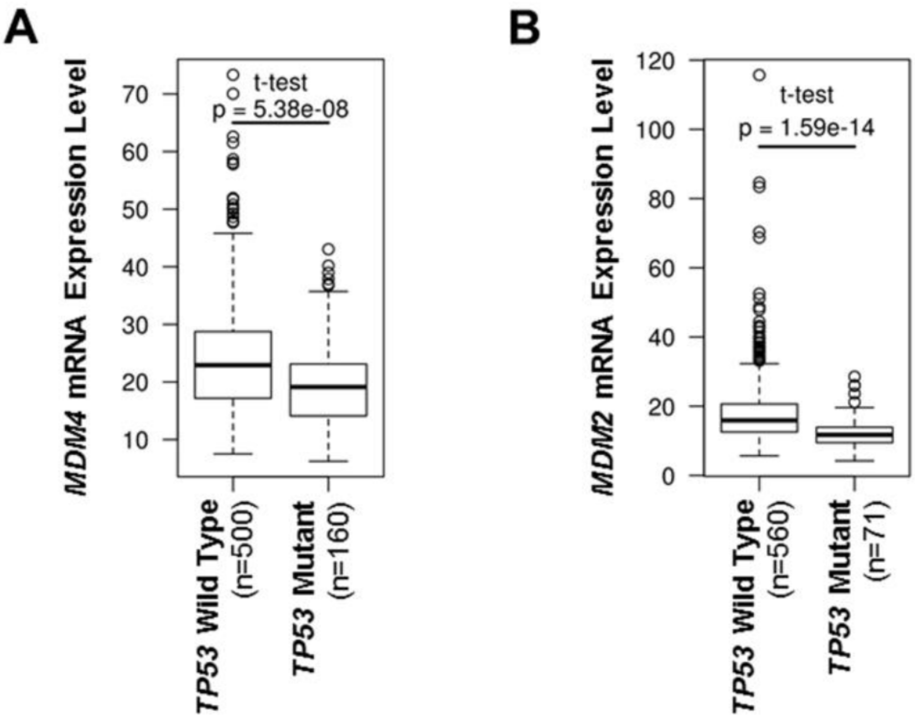

Supplementary Figure S1: (**A, B**) Analyses of MDM4 (panel A) or MDM2 (panel B) mRNA expression in TCGA breast invasive carcinoma cohort. Tumor types were classified according to *TP53* mutation status. To study gene expression patterns that were independent of *MDM4* or *MDM2* gene amplification, note that for panel A, samples with *MDM4* gene amplifications were excluded from the analysis, and for panel B, samples with *MDM2* gene amplifications were excluded from the analysis.

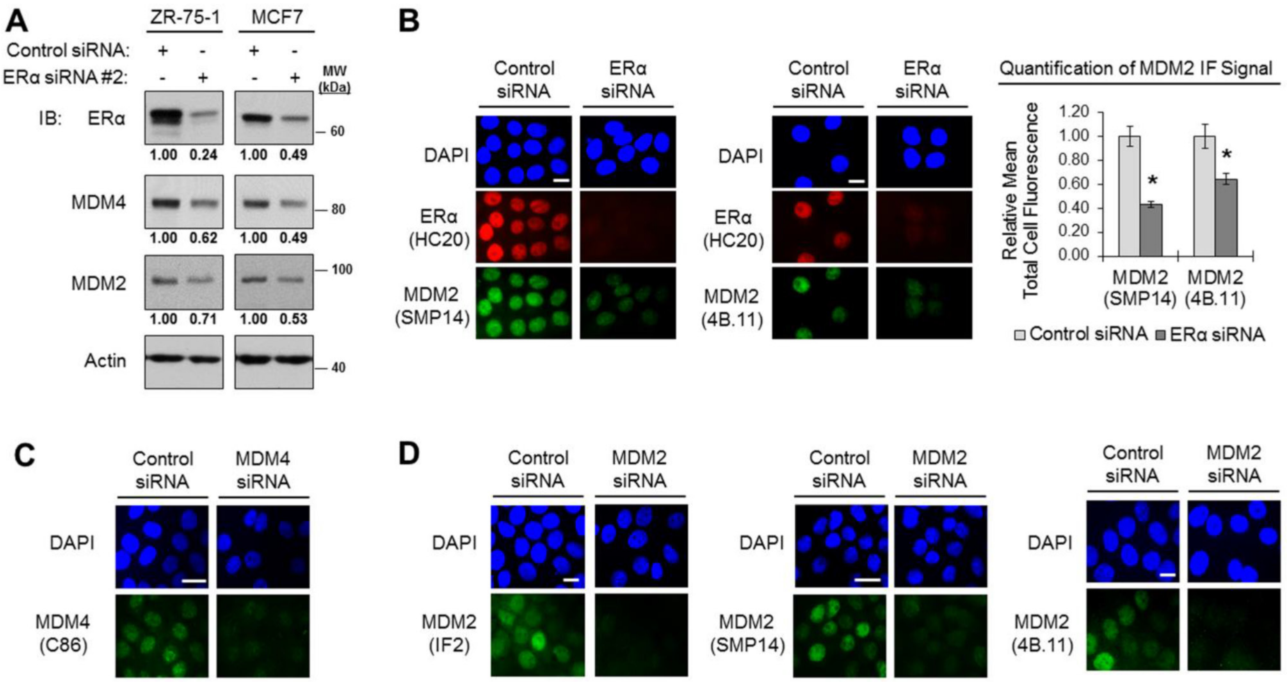

Supplementary Figure S2: (**A**) Western blot analysis of ZR-75-1 and MCF7 cells that were transfected with control siRNA or ERα siRNA (sequence #2) for 22 h. Numbers underneath the blot are the quantification of the relative band intensity, normalized to actin. (**B**) Immunofluorescent (IF) microscopy of ERα (red) and MDM2 (green) in MCF7 cells that were transfected with control siRNA or ERα siRNA for 22 h. Nuclei were labeled with DAPI (blue). Exposure times were equal for control siRNA- and ERα siRNA-transfected cells. Two different MDM2 antibodies were used for IF: SMP14 (left panel) and 4B.11 (center panel). Left and center panels: representative IF images. Right panel: quantification of IF signal (n = 15 cells from 3 independent coverslips). Error bars = SEM of total cell fluorescence. (**C**) IF microscopy of MDM4 (green) in MCF7 cells that were transfected with control siRNA or MDM4 siRNA for 48 h. Nuclei were labeled with DAPI (blue). Exposure times were equal for control siRNA- and MDM4 siRNA-transfected cells. (**D**) IF microscopy of MDM2 (green) in MCF7 cells that were transfected with control siRNA or MDM2 siRNA for 48 h. Nuclei were labeled with DAPI (blue). Exposure times were equal for control siRNA- and MDM2 siRNA-transfected cells. Three different MDM2 antibodies were used: IF2 (left panel), SMP14 (center panel) and 4B.11 (right panel). Microscopy scale bar = 20 µm. DAPI = 4′,6-diamidino-2-phenylindole. IB = immunoblot. kDa = kilodaltons. MW = molecular weight.

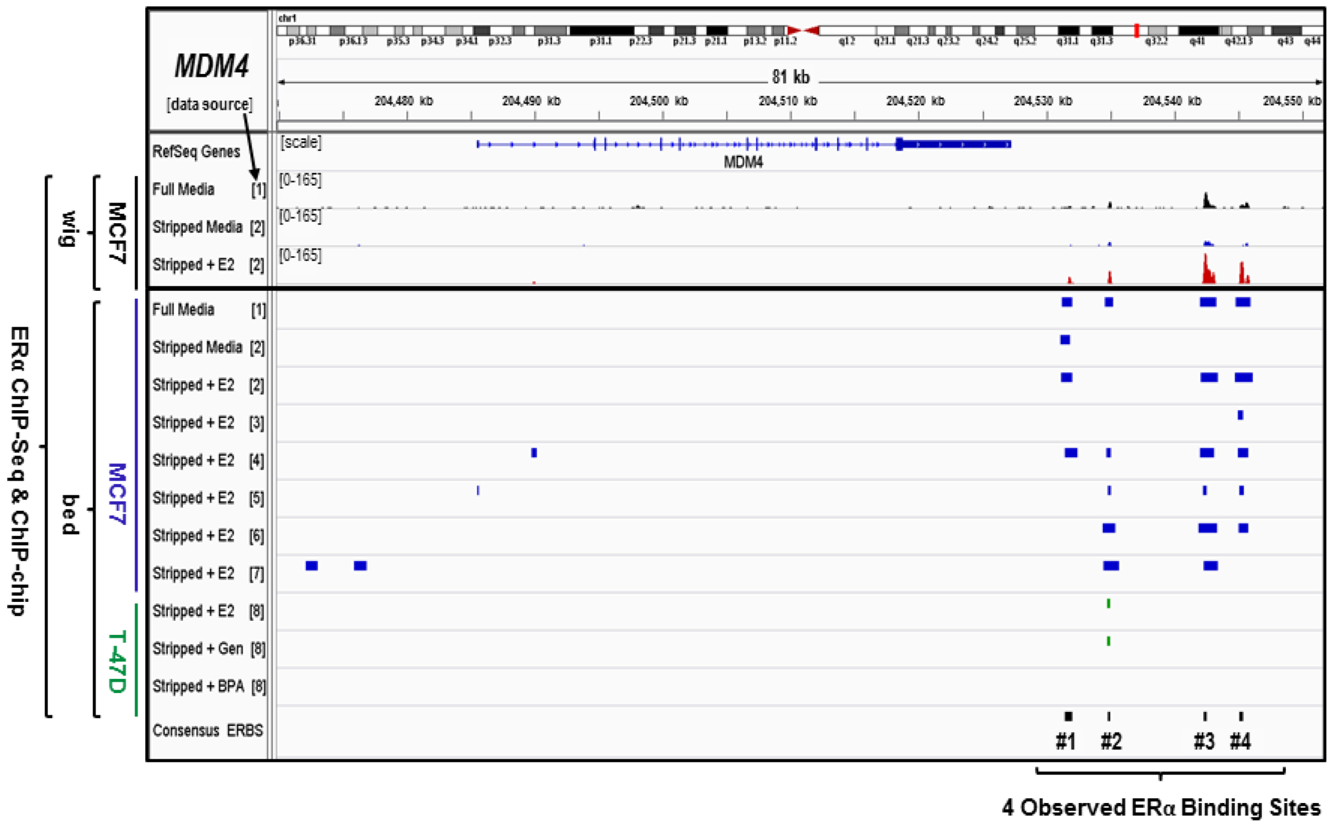

Supplementary Figure S3: IGV snapshot of ERα ChIP-Seq/Chip signal at the *MDM4* locus, as assessed by analyzing 8 different publicly available datasets. Upper panel: Individual tracks are wig files, representing ERα ChIP signal, from MCF7 cells that were cultured in full media (black), charcoal-stripped media (blue), or charcoal-stripped media containing 17β-estradiol (Stripped +E2; red). Lower panel: Individual tracks are bed files representing normalized ERα ChIP signal from MCF7 cells (blue) or T47D cells (green) that were cultured in full media, charcoal-stripped media, charcoal-stripped media containing one of three ERα agonists: 17β-estradiol (Stripped + E2), genistein (Stripped + Gen), or bisphenol A (Stripped + BPA). Four regions were observed to be bound by ERα in at least 2 or more of the 8 datasets and were subsequently named "consensus ERα binding sites (ERBS)" #1–4. These consensus ERBS are shown as bed files in black at the bottom of the figure. Data sources for Supplementary Figure S2: The following publicly available datasets for the MCF7 cells were downloaded from the Nuclear Receptor Cistrome database (http://cistrome.org/NR_Cistrome/), as deposited by the following investigators: [1] Antoni Hurtado, [2] Edison Liu, [3] Hendrik Stunnenberg, [4] Duncan Odom, [5] Arul Chinnaiyan, [6] Myles Brown, and [7] Kevin White. The following publicly available dataset for the T-47D cells was downloaded from the ENCODE Project database (https://www.encodeproject.org/): [8] Richard Meyers, ENCODE Consortium. Additional information about these datasets can be found in the Materials and Methods section.

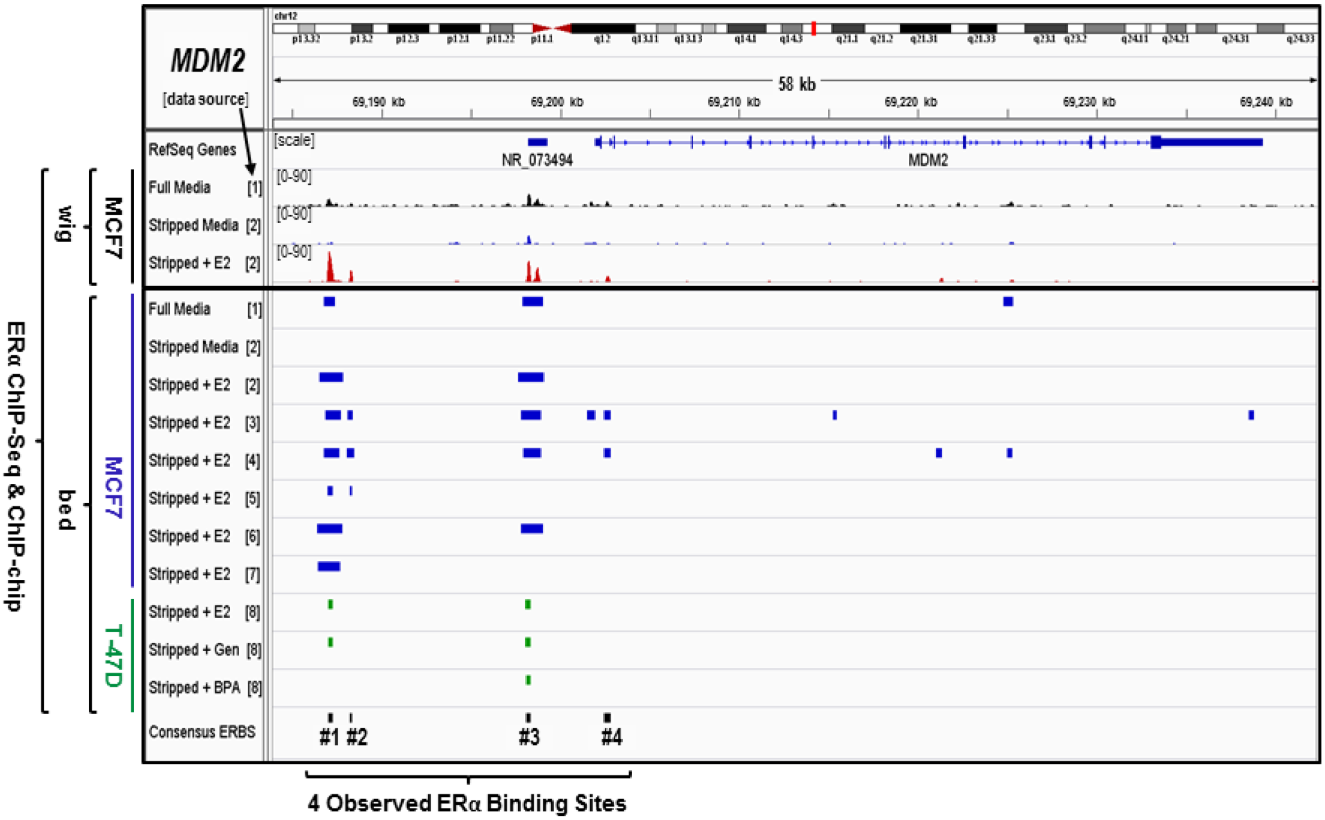

Supplementary Figure S4: IGV snapshot of ERα ChIP-Seq/Chip signal at the *MDM2* locus, as assessed by analyzing 8 different publicly available datasets. Upper panel: Individual tracks are wig files, representing ERα ChIP signal, from MCF7 cells that were cultured in full media (black), charcoal-stripped media (blue), or charcoal-stripped media containing 17β-estradiol (Stripped +E2; red). Lower panel: Individual tracks are bed files representing normalized ERα ChIP signal from MCF7 cells (blue) or T47D cells (green) that were cultured in full media, charcoal-stripped media, charcoal-stripped media containing one of three ERα agonists: 17β-estradiol (Stripped + E2), genistein (Stripped + Gen), or bisphenol A (Stripped + BPA). Four regions were observed to be bound by ERα in at least 2 or more of the 8 datasets and were subsequently named "consensus ERα binding sites (ERBS)" #1–4. These consensus ERBS are shown as bed files in black at the bottom of the figure. Data sources for Supplementary Figure S2: The following publicly available datasets for the MCF7 cells were downloaded from the Nuclear Receptor Cistrome database (http://cistrome.org/NR_Cistrome/), as deposited by the following investigators: [1] Antoni Hurtado, [2] Edison Liu, [3] Hendrik Stunnenberg, [4] Duncan Odom, [5] Arul Chinnaiyan, [6] Myles Brown, and [7] Kevin White. The following publicly available dataset for the T-47D cells was downloaded from the ENCODE Project database (https://www.encodeproject.org/): [8] Richard Meyers, ENCODE Consortium. Additional information about these datasets can be found in the Materials and Methods section.

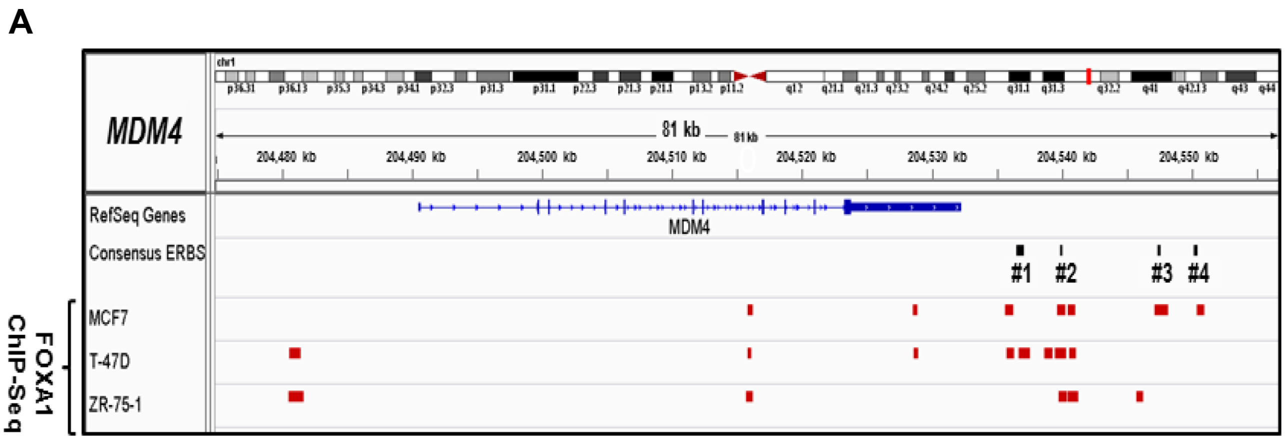

Supplementary Figure S5: IGV snapshots of the *MDM4* gene (**A**) and the *MDM2* gene (**B**), and analysis of publicly-available FOXA1 ChIP-Seq datasets. Individual tracks are bed files representing normalized FOXA1 ChIP-Seq signal from asynchronous MCF7, T47D, or ZR-75-1 human breast cancer cells that were cultured in full media. The ChIP-Seq datasets were downloaded from the Jason Carroll lab website (http://www.carroll-lab.org.uk/data); additional information about these datasets can be found in the Materials and Methods section. The consensus ERα binding sites (ERBS) identified from ERα ChIP in Supplementary Figures S2 and S3 are shown as black bed files.

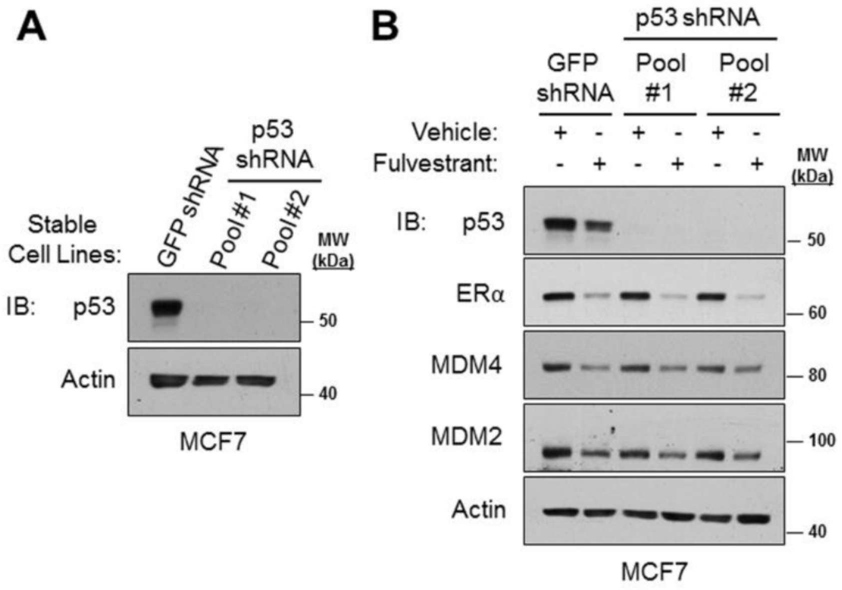

Supplementary Figure S6: (**A**) Western blot analysis of MCF7 cells that stably overexpress GFP shRNA or p53 shRNA (two different cell pools). (**B**) Western blot analysis of cells from panel A that were treated with vehicle (ethanol) or fulvestrant (1 μM) for 22 h. IB = immunoblot. kDa = kilodaltons. MW = molecular weight.

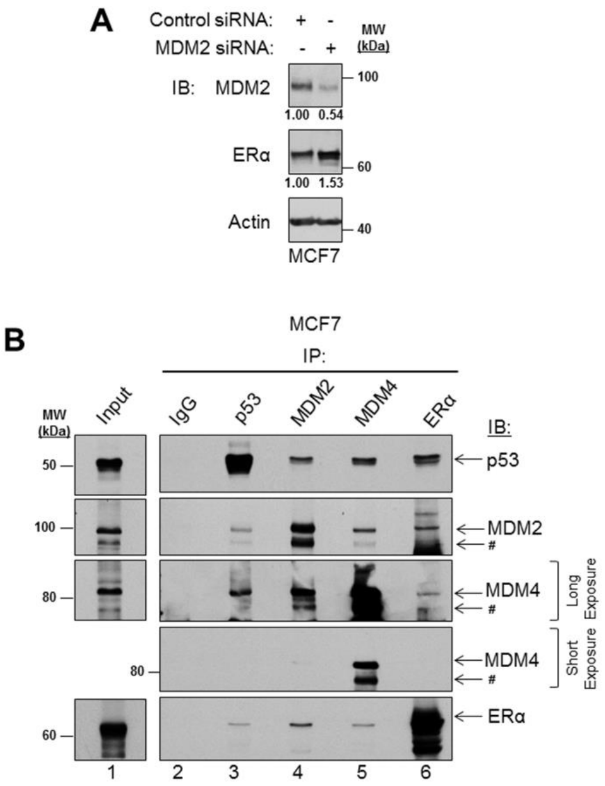

Supplementary Figure S7: (**A**) Western blot analysis of MCF7 cells that were transfected with control siRNA or MDM2 siRNA for 48 h. Numbers underneath the blot are the quantification of the relative band intensity, normalized to actin. (**B**) Co-immunoprecipitation (co-IP) analysis of protein complexes in MCF7 cell lysates. Immunoprecipitation (IP) was performed with normal IgG, p53 antibody, MDM2 antibody, MDM4 antibody or ERα antibody, as labeled above the blot. Immunoblot (IB) detection was performed using p53, MDM2, MDM4 or ERα antibodies, as labeled to the right of the blot. IgG is shown as a negative control. Interactions between p53-MDM2, p53-MDM4 and p53-ERα are shown as positive controls. # = related isoform, protein fragment or non-specific band. IB = immunoblot. IgG = immunoglobulin G. IP = immunoprecipitation. kDa = kilodaltons. MW = molecular weight.

Supplementary Table S1: Estrogen response elements (EREs) and Half-Sites in *MDM4*

ERBS[1]	EREs and Half-Sites[2]
#1	GAGCA gtc CGACC
#2	TGACC<br>TGACC<br>TGACC<br>GGTCA
#3	GGACA gcc TGACC<br>ATTCA ctt TGACC
#4	GAGCA gtg TGTCC<br>GGACA gtg TAACC
Canonical ERE	GGTCA nnn TGACC

[1]Estrogen Receptor Binding Sites (ERBS) #1–4 within the *MDM4* gene, as illustrated in Supplementary Figure S2.
[2]As predicted using Dragon ERE Finder Software.
Underlined letters indicate an imperfect ERE sequence, when compared to the canonical ERE sequence

Supplementary Table S2: Estrogen response elements (EREs) and Half-Sites in *MDM2*

ERBS[1]	EREs and Half-Sites[2]
#1	GAACA gat TGACC<br>GACCA tgc CGACC
#2	GGACA tcc TGACC
#3	GCCCA ctg TGACC
#4	GGTCC gga TGATC
Canonical ERE	GGTCA nnn TGACC

[1]Estrogen Receptor Binding Sites (ERBS) #1-4 within the *MDM2* gene, as illustrated in Supplementary Figure S3.
[2]As predicted using Dragon ERE Finder Software.
Underlined letters indicate an imperfect ERE sequence, when compared to the canonical ERE sequence.
